# Investigating the Overall Experience of Wearable Robots during Prototype-Stage Testing

**DOI:** 10.3390/s22218367

**Published:** 2022-11-01

**Authors:** Jinlei Wang, Suihuai Yu, Xiaoqing Yuan, Yahui Wang, Dengkai Chen, Wendong Wang

**Affiliations:** 1 Key Laboratory of Industrial Design and Ergonomics, Ministry of Industry and Information Technology, Northwestern Polytechnical University, Xi’an 710072, China; 2 Shaanxi Engineering Laboratory for Industrial Design, Northwestern Polytechnical University, Xi’an 710072, China; 3 School of Mechanical Engineering, Northwestern Polytechnical University, Xi’an 710072, China; 4 School of Design and Arts, Beijing Institute of Technology, Beijing 100811, China

**Keywords:** attitude, PLS-SEM, usability, user experience, WRs

## Abstract

Wearable robots (WRs) might interact with humans in a similar manner to teammates to accomplish specific tasks together. However, the available data on WR user experience (UX) studies are limited, especially during the prototyping phase. Therefore, this study aims to examine the overall experience of WRs during the prototyping phase based on an exploratory research model. This theoretical model considered usability, hedonic quality, and attitude toward using WRs as key factors in explaining and predicting overall experience. To test the hypotheses inherent in the research model, quantitative empirical research was conducted and the data were analyzed by partial least squares structural equation modeling (PLS-SEM). The results from the PLS-SEM analysis revealed the significance level of correlations between the latent variables in the research model. The exploratory research model was able to explain up to 53.2% of the variance in the overall experience of using WRs, indicating medium predictive power. This research develops a new quantitative empirical research model that can be used to explain and predict the overall experience of interactive products such as WRs. Meanwhile, the model is needed during WR testing in the prototype phase.

## 1. Introduction

Advances in robotic technology continue to replace human repetitive labor and expand the scope of human labor in areas of industrial production and daily life. For example, WRs can play an important auxiliary role in industrial production [1]. WRs, in a similar manner to teammates, also offer many potential benefits, which include preventing workers from acquiring musculoskeletal disorders (MSDs), improving operational accuracy, reducing labor intensity, saving labor time, and increasing endurance [1,2]. Although the unexpected increase in work-related MSDs has inspired researchers to explore the applicability of WRs for industrial workers to reduce medical visits [3,4,5], gaining these potential benefits of WRs is inseparable from assessments of usability and UX.

Assessments of usability and UX are often applied in the development of interactive products such as WRs. In order to achieve high product quality, UX imposes new usability requirements that drive the development of usability. Usability and UX can also play a significant role in the success of new interactive products [6]. Moreover, the potential benefits of usability and UX have been widely referenced for a range of purposes [2,7], including improving product performance, enhancing product popularity, increasing product sales, and expanding product marketization. For example, developers can add new features to the product based on the usability rating for improving product performance and designers can choose the right product surface material based on a UX score to enhance product popularity. In addition, sellers can set up a product-display environment based on UX score to increase product sales. However, in order to enjoy these benefits, users must first choose the desired product. The availability of the desired product does not guarantee that the desired product will sell well in the market. Therefore, we must explain and predict whether overall experience of interactive products meets users’ expectations. Despite the importance of usability and UX of interactive products such as WRs, very little research has extended UX models for the development of WRs.

Many companies have created WRs that can be used in a variety of application scenarios. For example, there are production scenes of material handling and decoration construction, daily scenes of going up and down stairs [8], as well as sports scenes of skiing and surfing. Moreover, the application of WRs in various production and life scenarios is still in its early stage. More workers may be willing to use WRs than housewives, since much of the work performed by workers is often extensive, repetitive, and arduous. However, developers do not pay enough attention to the UX of WRs, especially during the prototyping phase [3]. Most previous studies on WRs focused on usability or intelligent control technology, but often neglected research on the UX of WRs. For the theoretical development of usability and UX of WRs, it is necessary to look for the constructs that influence the overall experience of workers with WRs during the WR prototyping phase. The current study addressed the following questions:

What constructs can be considered as key factors to predict the overall experience of workers with WRs during the WR prototyping phase?

What are the relationships between the key factors?

## 2. Theories and Research Method

In the field of ergonomics, the concepts of usability and UX have gained attention in recent years. Moreover, companies have paid more attention to the value of usability and UX for an end product and invented marketing strategies based on them to sell products. Additionally, users interacting with the industrial products have different experiences, which can determine whether the industrial product can attract them and whether customers will use it again. Therefore, usability and UX are considered to be key factors determining the quality of the interactive products intended for human use, which in turn can be seen as indicators of the success or failure of the interactive products [6]. Assessments of usability and UX extensively facilitate the development of interactive products and considerably improve the quality of the interactive products. At the same time, the related concepts of usability and UX are inconsistent in academic communities and among practitioners, which may cause confusion.

### 2.1. Usability

The well-known standard definition of usability is proposed by International Standards Organization (ISO) [9]: “The extent to which a product can be used by specified users to achieve specified goals with effectiveness, efficiency and satisfaction in a specified context of use”, which is consistent with definition of latest version published by ISO [10]. The definition has been applied and developed in many publications. For example, a system usability scale (SUS) [11] with 10 items is the main non-invasive and low-cost usability measurement method reflecting the implication of this definition. Similarly, a usability metric for user experience (UMUX) with 4 items [12] corresponds to satisfaction, effectiveness, and efficiency of SUS. Afterwards, on the basis of UMUX, UMUX-LITE [13] was developed. A more recent study [14] evaluated the reliability of UMUX-LITE with different response options. The development of these usability theories not only promotes the improvement of the quality of interactive products, but also provides a reference for UX.

### 2.2. User Experience

The ISO [15] published the first formal definition of user experience (UX): “A person’s perceptions and responses that result from the use or anticipated use of a product, system or service”, which is consistent with the latest version of the definition provided by ISO [10]. The definition also emphasizes that usability is closely related to UX, which is in line with the views of many scholars [16,17]. Moreover, most practitioners and researchers consider UX to be a dynamic, context-dependent construct, and subjective [16,17,18]. For the application and measurement of UX, it referenced definition and studies of usability before the draft version of the definition of UX was published. For example, AttrakDiff 2 [19], as a simple and immediate way to measure UX, is driven from usability. A user experience questionnaire (UEQ) [20] was then designed as a reliable approach to measure UX. Furthermore, the constructs of UEQ are significantly related to the constructs of AttrakDiff2 [20]. Specifically, perspicuity, efficiency and dependability in UEQ correspond to the pragmatic quality aspects of AttrakDiff, while stimulation and novelty in UEQ correspond to hedonic quality aspects of AttrakDiff. Afterwards, the short version of the UEQ, which focuses on measuring the two meta-dimensions of the pragmatic and hedonic quality of UX, has been suggested as appropriate for certain scenarios [21]. In short, it is generally accepted that UX involves two meta-dimensions of pragmatic and hedonic quality in applying and measuring UX.

### 2.3. Overall Experience

Overall experience is the global responses or evaluation outcomes of users of a product after using it, which have positive correlations with usability, pragmatic quality, and hedonic quality [22,23]. Similarly, some studies [24,25] suggest that affect and usability can significantly predict overall experience. However, it might be more valuable if they examined whether there is an indirect effect of hedonic quality between pragmatic quality and overall experience. Researchers [25,26,27] measure overall experience with a 3-item scale (recommend product, use again, stimulating experience) or/and 4-item scale (motivated, recommend, enjoyable, satisfied), while Lewis [14] measure it with a single-item scale.

### 2.4. Attitude

Attitude refers to the extent to which a person feels positive or negative about performing a specific behavior. Furthermore, attitudes towards using products are determined by perceptions of the usefulness and ease of use [28], which are important aspects of perceived usability. Additionally, a positive attitude towards using the product relates to satisfaction as one of the items of overall experience [26].

### 2.5. Research Model and Hypotheses

In terms of constructs in this study, usability is the same as ergonomic quality or pragmatic quality in UX research [25,29,30]. To a certain extent, usability can completely replace pragmatic quality as part of UX [6,18,20,30,31], because usability almost overlaps with pragmatic quality [18,25], which is a key element of UX [21,30,32]. Likewise, hedonic quality, as another key element of UX, expands the pure usability perspective to touch the user emotionally, which can be predicted by usability [25,26,33]. Furthermore, usability mainly involves aspects of perceived usefulness and perceived ease of use [34], which is mediated by attitude-to-use in the technology-acceptance model, which means usability should correlate with attitude. However, whether attitude is able to predict overall experience and the hedonic quality of using an interactive product is not clear.

**Figure 1:**
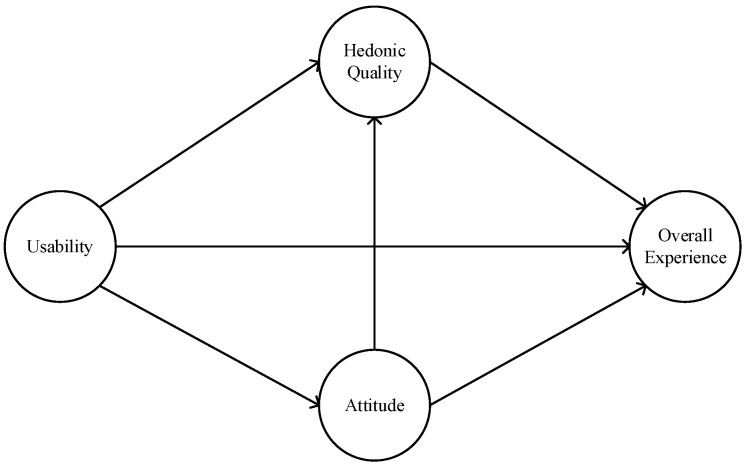
Research model.

From the above analysis, we consider usability, hedonic quality, attitude, and overall experience of using WRs as key elements to explore the science behind usability and UX. Unfortunately, no attempt has been made to quantify the association between usability, hedonic quality, attitude and overall experience of using WRs. Therefore, we develop an exploratory research model (see Figure 1) and propose the following hypotheses:

**H1a.**  *Usability has a significant direct effect on overall experience.*

**H1b.**  *Usability has a significant direct effect on hedonic quality.*

**H1c.**  *Usability has a significant direct effect on attitude.*

**H2a.**  *Attitude has a significant direct effect on overall experience.*

**H2b.**  *Attitude has a significant direct effect on hedonic quality.*

**H2c.**  *Attitude has a significant mediating effect on the effect between usability and overall experience.*

**H2d.**  *Attitude has a significant mediating effect on the effect between usability and hedonic quality.*

**H2e.**  *Attitude and hedonic quality have a significant mediating effect on the effect between usability and overall experience.*

**H3a.**  *Hedonic quality has a significant direct effect on overall experience.*

**H3b.**  *Hedonic quality has a significant mediating effect on the effect between usability and overall experience.*

**H3c.**  *Hedonic quality has a significant mediating effect on the effect between attitude and overall experience.*

## 3. Methods

### 3.1. Participants

This study collected data based on a nonrandom sampling technique that is convenient to sample. Inclusion criteria were 20–50 years old, male, and height 160–185 cm. We decided to include only males, because male workers dominate architectural decoration industries and manufacturing industries in China. A total of 152 healthy participants (26.6 ± 5.2 years) were recruited from the city Xi’an in western China, and they had no experience with WRs at all. They worked in courier services, supermarkets, or on construction sites. We reported data from 149 participants, as 3 participants were removed because they had no experience of using cordless screwdrivers or matching issues with the wearable robot. All participants voluntarily signed informed consents before taking part in the experiment. The study protocols, procedures, and consent form were approved by Medical and Experimental Animal Ethics Committee of Northwestern Polytechnical University (approbation number: 6101030222595-202001001).

### 3.2. Wearable Robot

**Figure 2:**
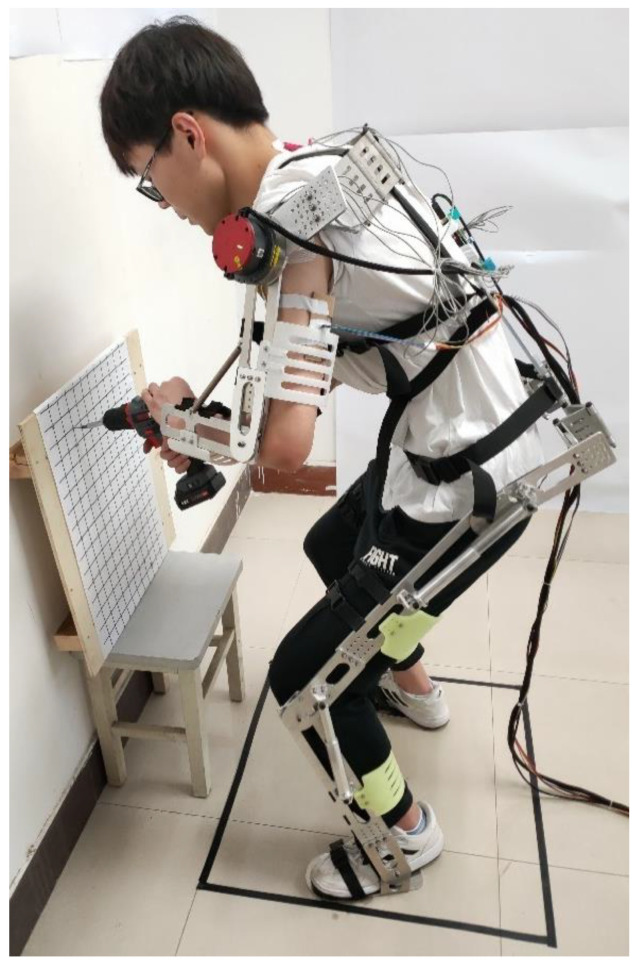
Experimental setup: Participant with wearable robot is using a cordless screwdriver to install screws in the requested location. Working height and distance to the set-up were individually adjusted to ensure a knee angle of ~45°—the installation and removal position.

The wearable robot (7.9 kg; see Figure 2) was developed by our lab, and can assist users with a height of 160–185 cm to carry heavy loads, to maintain a balanced posture, and improve operation accuracy. It consists of arm bounds, leg bounds, foot bindings, and four adjustable limbs. The two upper limbs with two motors give the user timely active assistance at the shoulder and elbow joints based on changes of emulsion signal. Additionally, the two lower limbs with four springs passively support the user at the hip and knee joints, and conduct the load from the upper limbs directly to the ground.

### 3.3. Experimental Procedure

We first introduced the experimental procedure and the wearable robot (see Figure 2) to the participants. In the second step, participants put on the wearable robot after adjusting the size to match their height. In the third step, the participant with the wearable robot found the exact positions on a wood board (550 × 550 × 12 mm) with a grid (30 × 30 mm) where the self-tapping screws would be drilled. In the fourth step, the participant installed 3 self-tapping screws (length: 15 mm) with a cordless screwdriver (weight: 1.48 kg). After installation, the participant needed to remove the 3 self-tapping screws. During installation and removal, the participants bent forward to maintain a half-squat position. As a final step, the participants filled out questionnaires (see Table 1).

### 3.4. Data Collection

The survey instrument contained 15 items derived from previous studies (see Table 1). Additionally, the survey measured usability, hedonic quality, attitude, and overall experience with a 5-point Likert scales ranging from “strongly disagree” to “strongly agree”. All data were collected in a paper version. Although the focus of the study is not to see if the items used in the study reflect all of the details of the four constructs, they must at least roughly measure constructs. Following the above experimental procedure, each participant completed the test for about 20 min. The participants could withdraw from the test at any time they wish. The entire process of data collection took 17 days. All 149 questionnaires distributed were recovered and qualified.

### 3.5. Data Analysis

The PLS-SEM has been used in the past to study the mechanical properties of latent variables and the relationships between different constructs. PLS-SEM has recently gained wide acceptance among research scholars [41,42,43] and has been widely adopted, including in WR studies [4]. This study is exploratory, which is one of the key reasons for choosing PLS-SEM for the analysis. PLS-SEM is suitable for small sample sizes, formative measures, non-normal data, theory development, and so on [44]. On the one hand, the sample size in this study is relatively small. On the other hand, the relationships between the four constructs are not explored in the literature. Besides this, there is no adequate theoretical basis explaining the relationships between these four constructs. These factors therefore make PLS the appropriate method for data analysis in this study. The evaluation of PLS-SEM by SmartPLS version 3 involves measurement-model evaluation and structural model evaluation in the path model, which follows Hair’s recommended systematic evaluation of PLS-SEM results [41,42]. In the process of the data analysis, raw data were imported directly into SmartPLS. The default settings of SmartPLS were used for measurement-model evaluation in this study. Specifically, we executed 10,000 subsamples in bootstrapping to derive the significance of relationships in structural model evaluation and applied the PLS_predict_ procedure with 10-fold cross-validation to access the predictive power of the structural model.

## 4. Results

### 4.1. Measurement Model Evaluation

The evaluation of internal consistency reliability of a measurement model should be based on Cronbach’s alpha (α) and composite reliability (ρ_C_). In addition, α forms the lower boundary of the internal consistency reliability, while ρ_C_ repents the upper boundary [41]. Besides this, researchers should also consider ρ_A_ as a suitable compromise between these two metrics [41,45,46]. Additionally, α, ρ_A_, and ρ_C_ have the same minimum limit (>0.7) of internal consistency reliability for evaluating reflective measurement models [45]. According to these criteria, Table 2 shows the values of α, ρ_A_, and ρ_C_, all of which are above 0.7 and meet the minimum limit. Moreover, the ρ_A_ values of attitude, hedonic quality, and usability lie between their α values and ρ_C_ values. However, the ρ_A_ value of overall experience is very close to the ρ_C_ value of overall experience, which is acceptable in exploratory research. Therefore, these results suggest that the construct measures of usability, attitude, hedonic quality, and overall experience exhibit appropriate levels of internal consistency reliability.

The convergent validity of the reflective measurement model is evaluated by loadings and average extracted variance (AVE) [45]. Generally speaking, all the loadings should be larger than 0.708, which shows that all items meet the basic requirements of reliability. Similarly, AVE as a key metric of convergent validity is expected to be above 0.5, suggesting that the construct explains more than 50 percent of the variance of its items [42,45]. Furthermore, all AVE values in Table 2 are larger than 0.50, indicating that all indicators have a sufficient level of reliability.

The assessment of discriminant validity should rely on the heterotrait-monotrait ratio (HTMT) instead of the two traditional approaches—the Fornell–Larcker criterion [47] and an examination of cross loadings—in applications of PLS-SEM [41,48]. Because of the homogeneousness of the indicator loadings, it is difficult for the Fornell–Larcker criterion to detect the discriminant validity of the reflective measurement model. In comparison, the cross loadings as an item-level discriminant performs worse than the Fornell–Larcker criterion in terms of validity [49,50]. Conversely, HTMT based on the multitrait-multimethod (MTMM) matrix performs better than these two traditional approaches in assessing discriminant validity [48]. Moreover, scholars [48,51] recommend 0.85 as the lower bound of HTMT values for comparing conceptually different constructs and 0.90 as the upper bound of HTMT values for comparing conceptually similar constructs. As a result, all HTMT values in Table 3 are all less than the conservative threshold of 0.85, which presents good discriminant validity for the reflective measurement model in the research model.

### 4.2. Structural Model Evaluation

The evaluation of the structural model is based on the acceptable quality of the reflective measurement model. Next, we need to check the significance and relevance of the relationships between endogenous and exogenous constructs, as well as the explanatory and predictive power of the research model. First, we must ensure that there is no bias in the regression results of path coefficients to avoid collinearity issues by checking the values of variance inflation factor (VIF). In practice, VIF values near 3 or below are ideal [52,53]. Therefore, all VIF values (see Table 4) among the four latent variables in the model are less than 3, indicating that there are no collinearity issues in the structural model.

Second, after verifying the potential collinearity issues among the four constructs, the size and significance of the path coefficients are assessed with respect to the correlations hypothesized between the constructs. The *t*-values, *p*-values, and confidence intervals of the path coefficients especially are computed by bootstrapping applied with 10,000 subsamples [54], because bootstrapping is a dependable and useful technique to identify null effects. When zero falls into the 95% percentile confidence interval, the path coefficient is not significant at the prespecified significance level [45]. Besides this, the percentile method is proposed to calculate confidence intervals because of its reliable effectiveness compared other methods [55].

We start with the direct effects when analyzing the path coefficient estimates of the structural model (see Table 5). Usability as the key predictor has different significant effects on attitude, hedonic quality, and overall experience. Usability has the strongest significant effect on attitude (0.497), followed by hedonic quality (0.336), and overall experience (0.255). Moreover, attitude has stronger significant effect on hedonic quality (0.263), and has a weak effect on overall experience (0.149), which is not significant at the 5% significance level. Similarly, hedonic quality has a strong significant effect on overall experience (0.480). When analyzing the specific indirect effects of attitude and hedonic quality, attitude mediates the relationship between usability and hedonic quality. Nevertheless, the 95% percentile confidence interval of U→ATT→OE contains zero, showing that attitude does not mediate the relationship between usability and overall experience. Conversely, hedonic quality mediates the relationship between attitude and hedonic quality and the relationship between usability and overall experience. Regarding the total effects on overall experience, usability has the strongest total effect (0.553), followed by hedonic quality (0.480), and attitude (0.275).

When examining the significance of the path coefficients of direct effects at significance level 5% (see Table 5), it was found that the hypothetical relationships U→OE, U→HQ, U→ATT, ATT→HQ, and HQ→OE are significant in the structural model, while ATT→OE is not. Therefore, the empirical results support Hypotheses 1a, 1b, 1c, 2b, and 3a, and lead us to reject Hypothesis 2a. Similarly to examination, we found that all path coefficients of specific indirect effects were significant except for U→ATT→OE. Therefore, we find empirical support for Hypotheses 2d, 2e, 3b, and 3c, and reject Hypothesis 2c.

**Figure 3:**
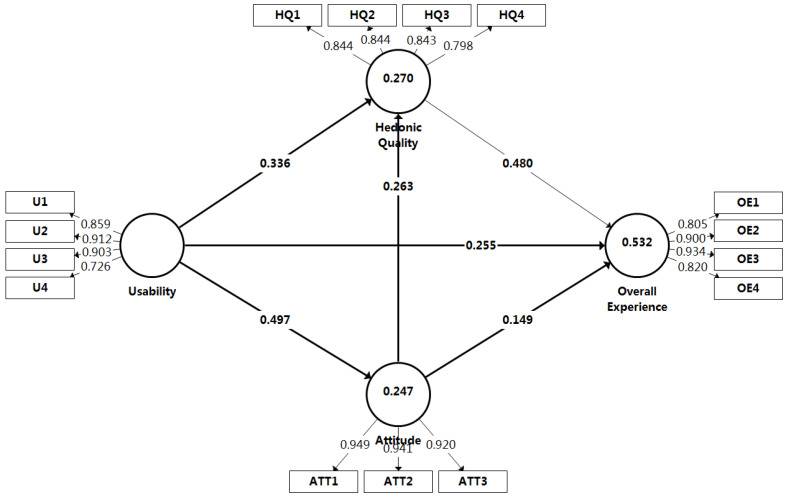
Path coefficients of the research model.

Third, the examination of the in-sample explanatory power of the research model is conducted with R^2^ measuring the variance explained in each of the endogenous constructs [42,56,57]. As a simple guide, although R^2^ values of 0.75, 0.50, and 0.25 can be considered substantial, moderate, and weak [58,59], R^2^ values of 0.1, depending on the research context, can be satisfactory [60]. In addition to R^2^ values of 0.9 or higher, this could signify reasonable model fit for a physical process rather than human intentions, perceptions, and attitudes [41,42]. According to this guide, the exploratory research model explains 53.2% of the overall experience (R^2^ = 0.532), indicting the explanatory power between substantial and moderate. See Figure 3; the R^2^ value of attitude is 0.247, and the R^2^ value of hedonic quality is 0.270, which are acceptable to satisfy the explanatory power of the exploratory research model.

Researchers can use the f^2^ effect size to assess how removing a particular predictor construct influences the R^2^ value of an endogenous construct. As a rule of thumb, f^2^ values above 0.02, 0.15, and 0.35 are considered small, medium, and large, respectively [61]. Additionally, f^2^ values below 0.02 indicate no effect is present. Table 6 demonstrates the f^2^ effect size. A relatively small f^2^ effect size occurs for the relationships ATT→OE (0.034), ATT→HQ (0.071), and a comparatively large f^2^ effect size occurs for the relationships U→AT (0.327), HQ→OE (0.360).

Finally, the examination of the out-sample predictive power of the research model is conducted with Q^2^_predict_ calculated by PLS_predict_ procedure [48,51], which adopts k-fold cross-validation. All Q^2^_predict_ values of items are above zero (see Table 7), which suggests that the predictive power of the PLS-SEM analysis for that all indicators outperforms the naïve benchmark. When comparing the root mean squared error (RMSE) values with the naïve linear regression model (LM) benchmark, the majority of RMSE values of items in the PLS-SEM analysis are less than the prediction errors in the LM analysis, which indicates a medium predictive power of the structural model.

## 5. Discussion

Although there is little comparable literature on UX for human–robot interaction that could be used as a confirming reference, our results described above are partially consistent with existing empirical studies on UX. In addition, the survey on the UX of wearable robots showed clear effects of usability, hedonic quality, and attitude on the overall user experience when using WRs. In contrast to the findings of [22,23,25], the largest direct effect was found for hedonic quality rather than usability. Nevertheless, users’ perceived usability plays an important role in predicting their overall experience of using WRs. Besides this, we did not find a direct effect of attitude on overall experience, which was out of line with our expectations and the result from Hart [26]. However, the 5.8% statistical result of the direct effect is very close to the 5% significance level, which may mean that further investigation is needed to verify the relationship between attitude and overall experience. Furthermore, the effect of usability can be partially explained by changes in hedonic quality and attitude. Therefore, additional explanations for the influence of these factors on overall experience in using WRs should be found. A possible explanation is social influence such as conformity [62], but this should be explored in future research.

Our results provided us with statistical support in answering our two research questions. In particular, the degree/strength of the relationships between usability, hedonic quality, attitude, and overall experience is reflected by the total effects and path coefficients in the structural models. Moreover, all total effects are positive and significant (see Table 5), which suggests that each correlation in this theoretical model reflects the level of users’ perceptions in relation to the usability and UX of WRs. Additionally, the total effects of U→OE (0.553), U→ATT (0.497), U→HQ (0.467), and HQ→OE (0.480) were sensitive, which implies that users’ perceived usability is an important predictor affecting their perceived hedonic quality and overall experience as well as attitude to using WRs. However, the total effects of ATT→HQ (0.263) and ATT→OE (0.275) were relatively insensitive, and the path coefficient of the direct effect of ATT→OE was not statistically significant.

### 5.1. Theoretical Implications

Based on previous studies, this study developed a new theoretical model where usability is considered similar to ergonomic/pragmatic quality as part of UX. We found that usability has the strongest total effect on the overall experience of using WRs in the research model, while hedonic quality has the strongest direct effect on overall experience of using WRs. In fact, users might care about hedonic quality if the usability of WRs meets users’ needs. In addition, the correlations between the four constructs can be referred to when researchers explore the science behind usability and UX for the implications of WRs. In summary, we do not claim to have used a standardized method for evaluating the overall experience of WRs. However, we believe that our investigation contributes to the implementation of WRs by providing useful insights into the usability and UX of WRs. In addition, this research model is exploratory and open-ended, so we are willing for more researchers to develop other theoretically reasonable models with different configurations and compare them according to model-selection criteria.

### 5.2. Practical Implications

The scales of the four constructs have statistical reliability and validity, which means these scales are appropriate to measure usability, hedonic quality, attitude, and overall experience of using WRs to support the assessment of new wearable technology. Additionally, the results suggest that users value product attributes related to hedonics more than those related to usability or pragmatics in order to create a better overall experience. This, in turn, implies that developers and designers should pay more attention to the hedonic quality of WRs by offering innovative WR shapes and interesting interactive modes that could attract more users to adopt WRs and recommend them to others. Moreover, WR companies can employ various strategies to gain support for WRs from their end users. For example, one of the strategies is to allow more potential users to try WRs for free in real-world scenarios to improve their perception of usability, especially hedonic quality, which can also influence attitudes towards using WRs. Additionally, a preliminary survey such as ours can provide an assessment of what potential users think.

### 5.3. Limitations

Although this research has some limitations, it creates a significant number of opportunities. First, we must note that our sample size for the PLS-SEM analysis was relatively small. In addition, all participants in our experiment were male, which might cause potential bias in this study. Therefore, future research data analysis should be based on large samples containing a reasonable number of female users. Second, in our case, all participants had no practical experience with WRs. We argue that it is also interesting for end-users to get the opinions of experienced users before actually making a purchase. After all, WRs are more expensive than general decorating tools such as cordless screwdrivers, so end-users must want to know how experienced users rate the purchase. Third, the wearable robot prototype used in the experiment has some obvious flaws. For example, modules such as batteries are not integrated together, which can inevitably affect portability. Future research should focus on overcoming these shortcomings and developing a mature product. Applying WRs to real-world environments will be part of our future research to gain insights into the overall experience of experienced users.

## 6. Conclusions

This study investigated the determinants affecting end users’ overall experience of WRs by developing an exploratory model. The results of PLS-SEM analysis indicated that the model explains 53.2% of the overall experience in using WRs and has medium predictive power for it. More specifically, usability has the strongest total effect on overall experience of using WRs in the research model, and hedonic quality has the strongest direct effect on the overall experience of using WRs. Although the direct effect of the attitude toward using WRs on overall experience was not statistically significant, attitude mediated the effect between usability and hedonic quality. Attitude and hedonic quality significantly mediated the effect between usability and overall experience. Hedonic quality significantly mediated the effect between usability and overall experience and the effect between attitude and overall experience. These results can provide empirical evidence to developers and designers in the prototyping phase of WRs to improve the hedonic quality of WRs or adapt to different application scenarios to meet different needs of potential users. When researchers or developers conduct similar or related studies of wearable technology, they can adopt the measurement models and structural model of this study. The results of this investigation have implications for quantitative and qualitative research on usability and UX, as well as the development and design of WRs.

## Figures and Tables

**Table 1:** Measurement properties of constructs.

Code	Items
Usability [3,12,23,35]	
U1	This wearable robot’s capabilities meet my requirements.
U2	Using this wearable robot enables me to operate accurately.
U3	This wearable robot is easy to use.
U4	Using this wearable robot enables me to accomplish tasks more quickly.
Hedonic quality [20,21,33,36,37]	
HQ1	I would feel interesting wearing the wearable robot.
HQ2	The wearable robot looks exciting to wear and use.
HQ3	Working with the wearable robot is original.
HQ4	It would be innovative for me to use the wearable robot at work.
Attitude [4,38,39]	
ATT1	Using the wearable robot is a good idea.
ATT2	Using the wearable robot in my coursework would be a pleasant experience.
ATT3	I like working with the wearable robot.
Overall experience [14,23,25,26,40]	
OE1	I feel motivated to continue to use the wearable robot.
OE2	I would recommend the wearable robot to my friends.
OE3	My experience of using the wearable robot is enjoyable.
OE4	Overall, I am very satisfied with the wearable robot.

**Table 2:** Assessment results of reliability and validity of measurement models.

Constructs	Items	Loadings	α	ρ_A_	ρ_C_	AVE
		>0.7	>0.7	>0.7	>0.7	>0.5
Attitude	ATT1	0.949	0.930	0.937	0.956	0.878
	ATT2	0.941				
	ATT3	0.920				
Hedonic Quality	HQ1	0.844	0.852	0.859	0.900	0.693
	HQ2	0.844				
	HQ3	0.843				
	HQ4	0.798				
Overall Experience	OE1	0.805	0.888	0.894	0.923	0.751
	OE2	0.900				
	OE3	0.934				
	OE4	0.820				
Usability	U1	0.859	0.875	0.907	0.914	0.728
	U2	0.912				
	U3	0.903				
	U4	0.726				

Notes: α = Cronbach’s alpha; ρ_A_ = rho_A; ρ_C_ = Composite Reliability; AVE = Average Variance Extracted.

**Table 3:** HTMT values of measurement model.

	Attitude	Hedonic Quality	Overall Experience	Usability
Attitude				
Hedonic quality	0.482			
Overall Experience	0.525	0.757		
Usability	0.529	0.519	0.605	

**Table 4:** VIF values of the structural model.

Constructs	Attitude	Hedonic Quality	Overall Experience
Attitude		1.327	1.422
Hedonic quality			1.370
Usability	1	1.327	1.482

**Table 5:** Significance testing results of the structural model path coefficients.

Direct Effects	O	M	STDEV	T	P	95% Confidence Interval
ATT→HQ	0.263	0.262	0.097	2.703	0.007	[0.070, 0.448]
ATT→OE	0.149	0.148	0.079	1.897	0.058	[−0.006, 0.448]
HQ→OE	0.480	0.481	0.061	7.812	0.000	[0.355, 0.597]
U→ATT	0.497	0.499	0.066	7.468	0.000	[0.361, 0.620]
U→HQ	0.336	0.340	0.088	3.802	0.000	[0.166, 0.511]
U→OE	0.255	0.255	0.079	3.211	0.001	[0.097, 0.406]
**Specific Indirect Effects**						
U→ATT→HQ	0.130	0.131	0.053	2.448	0.014	[0.033, 0.242]
U→ATT→OE	0.074	0.075	0.042	1.746	0.081	[−0.003, 0.166]
ATT→HQ→OE	0.126	0.127	0.051	2.49	0.013	[0.031, 0.230]
U→ATT→HQ→OE	0.063	0.063	0.027	2.286	0.022	[0.015, 0.122]
U→HQ→OE	0.162	0.163	0.047	3.418	0.001	[0.075, 0.261]
**Total Effect**						
ATT→HQ	0.263	0.262	0.097	2.703	0.007	[0.070, 0.448]
ATT→OE	0.275	0.274	0.092	2.996	0.003	[0.089, 0.451]
HQ→OE	0.480	0.481	0.061	7.812	0.000	[0.355, 0.597]
U→ATT	0.497	0.499	0.066	7.468	0.000	[0.361, 0.620]
U→HQ	0.467	0.472	0.067	6.998	0.000	[0.332, 0.595]
U→OE	0.553	0.557	0.064	8.658	0.000	[0.425, 0.674]

Note. ATT = attitude; HQ = hedonic quality; U = usability; OE = overall experience; O = original sample; M = sample mean; STDEV = standard deviation.

**Table 6:** Values of f^2^.

	f^2^	Category
U→ATT	0.327	Large
U→HQ	0.117	Moderate
U→OE	0.094	Small
ATT→HQ	0.071	Small
ATT→OE	0.034	Small
HQ→OE	0.360	Large

**Table 7:** PLS_predict_ results.

Items	PLS		LM	PLS-LM
	RMSE	Q^2^_predict_	RMSE	RMSE
ATT1	1.028	0.232	1.030	−0.002
ATT2	1.097	0.220	1.095	0.002
ATT3	1.114	0.162	1.134	−0.020
HQ1	1.135	0.130	1.155	−0.020
HQ2	1.052	0.221	1.066	−0.014
HQ3	1.086	0.103	1.099	−0.013
HQ4	1.066	0.088	1.080	−0.014
OE1	1.006	0.169	1.008	−0.002
OE2	1.036	0.197	1.045	−0.009
OE3	0.921	0.259	0.918	0.003
OE4	0.935	0.253	0.921	0.014

## Data Availability

The data presented in this study are available on request from the corresponding author.
